# Predicting metabolic syndrome in adolescents from device-measured 24-h movement behaviors: a machine learning approach

**DOI:** 10.3389/fpubh.2026.1928562

**Published:** 2026-09-17

**Authors:** Liquan Cao, Zhanpeng Feng, Jing Wang, Yun Wang

**Affiliations:** 1 Tianjin University of Sport, Tianjin, China; 2 Tianjin Key Laboratory of Sports and Health Integration and Health Promotion, Tianjin, China

**Keywords:** metabolic syndrome, adolescent, 24-hour movement behaviors, machine learning, SHAP

## Abstract

**Objective:**

To develop and internally evaluate a non-invasive machine-learning model for identifying metabolic syndrome (MS) in adolescents using 24-h movement behaviors and non-invasively measured physical characteristics.

**Methods:**

This cross-sectional study included 822 adolescents aged 12–15 years recruited in Tianjin, China, from September 2023 to September 2025. Movement behaviors were assessed using ActiGraph GT3X+ accelerometers, body composition was measured using dual-energy X-ray absorptiometry, and MS was diagnosed according to the 2007 International Diabetes Federation criteria. LASSO regression was used for feature selection, and 13 machine-learning algorithms were compared. SMOTETomek was used during model development to address class imbalance. Performance was evaluated using stratified five-fold cross-validation and a held-out internal test set. SHapley Additive exPlanations (SHAP) were used to interpret model predictions.

**Results:**

LASSO retained eight predictors: waist circumference, body fat percentage (FAT%), weight, age, moderate physical activity (MPA), vigorous physical activity, sleep duration, and sex. Extra Trees achieved the best cross-validation performance, with a mean AUC of 0.749 (SD, 0.043) and an F1 score of 0.798 (SD, 0.028). Its relative advantage may reflect the ability of randomized tree ensembles to capture nonlinear relationships and feature interactions while reducing variance through aggregation. In the held-out internal test set, the model achieved an AUC of 0.864, accuracy of 92.02%, specificity of 93.33%, and sensitivity of 76.92%. The lower sensitivity relative to accuracy and specificity indicates that some adolescents with MS may remain undetected. SHAP ranked FAT% as the most influential predictor (mean absolute SHAP value, 0.08) and MPA as the leading movement-behavior predictor (0.04), thereby improving the transparency of model predictions.

**Conclusions:**

The Extra Trees model showed moderate cross-validation discrimination and stronger performance in the internal test set, although this difference warrants cautious interpretation. SHAP improved model transparency by identifying FAT% and MPA as influential predictors. Multicenter external validation and feasibility assessment are required before large-scale screening.

## 1 Introduction

Metabolic syndrome (MS) is a clinical syndrome characterized by the clustering of central obesity, impaired glucose regulation, hypertension, and dyslipidemia (1), and represents a major risk factor for type 2 diabetes mellitus (T2DM) (2) and cardiovascular disease (CVD) (3). In recent years, with the persistently rising global prevalence of childhood and adolescent obesity, the prevalence of adolescent MS has also shown a rapid upward trend, emerging as a critical public health concern affecting youth health (4, 5). According to the International Diabetes Federation (IDF) 2007 criteria, the global prevalence of MS among adolescents ranges from 3% to 30%, with substantial disparities between developed and developing regions (6); among overweight and obese adolescents, this proportion reaches 15%−25%, and overweight/obesity constitutes a significant risk factor for MS (7, 8). MS not only markedly elevates the risk of glucolipid metabolic abnormalities and hypertension during adolescence (9) but is also closely associated with elevated CVD events and all-cause mortality in adulthood (10). Therefore, early identification of high-risk adolescents and implementation of targeted interventions are of considerable importance for reducing the lifetime CVD burden (11).

The World Health Organization (WHO) 2020 Guidelines on Physical Activity and Sedentary Behavior (12) explicitly state that 24-h activity behaviors comprise four core components—sleep (SLP), sedentary behavior (SB), light-intensity physical activity (LPA), and moderate-to-vigorous physical activity (MVPA)—among which time allocation is subject to a total-time constraint (13). Dumuid et al. (14) first provided a statistical framework in 2017 for quantifying the effects of reallocating time among these components on health outcomes, which has since been widely applied in research on physical activity and metabolic health (15, 16). A systematic review by Carson et al. (17) confirmed that insufficient MVPA and excessive SB are significantly associated with individual MS components in adolescents, including elevated triglycerides (TG), reduced high-density lipoprotein cholesterol (HDL-C), and elevated blood pressure.

The rapid advancement of machine learning methods in medical prediction modeling has provided new technical tools for the early screening of complex metabolic phenotypes such as MS (18), enabling the effective capture of non-linear interaction effects among features (19). Zhang et al. (20), using data from the National Health and Nutrition Examination Survey (NHANES), demonstrated that machine learning models achieved high predictive performance (AUC = 0.85) for adolescent MS using non-invasive indicators, suggesting the feasibility of large-scale non-invasive screening. However, existing machine learning studies on adolescent MS prediction have yet to fully integrate the multi-dimensional characteristics of 24-h activity behaviors and lack in-depth SHAP-based interpretation of model decision mechanisms (21). Accordingly, the present study aims to construct a machine learning model for adolescent MS that integrates 24-h activity behaviors with anthropometric indicators, providing a scientific basis for early screening and informing the development of precision intervention strategies based on activity behavior modification.

## 2 Methods

### 2.1 Participants and study design

This study employed a cross-sectional survey design. Sample size estimation was performed using the formula method proposed by Hsieh et al. (22), with α = 0.05 (two-tailed), statistical power (1–β) = 0.80, and an anticipated effect size (odds ratio) of 2.0, yielding a minimum required sample size of 250 participants. The study was conducted in Tianjin, China from September 2023 to September 2025. A stratified cluster sampling strategy was adopted, randomly selecting four public junior high schools and four public senior high schools. Within each school, all eligible students in the selected grade were enrolled as a cluster. After applying the inclusion and exclusion criteria, a total of 822 participants were enrolled, comprising 385 males (46.8%) and 437 females (53.2%), with an age range of 12–15 years (mean age 13.18 ± 0.98 years). The dataset was partitioned into a training set and a test set at an 8:2 ratio (23). Inclusion criteria: (1) aged 12–15 years with normal physical development; (2) free of severe organic diseases of the heart, lungs, liver, or kidneys; (3) voluntary participation with signed informed consent. Exclusion criteria: (1) diagnosed conditions affecting physical growth and development, such as dwarfism or gigantism; (2) acute infectious disease, fever, or diarrhea within the preceding two weeks; (3) use of medications that may affect metabolic indicators within the preceding 3 months, such as glucocorticoids or insulin; (4) inability to complete all test items. This study was approved by the Ethics Committee of Tianjin University of Sport (Approval No.: TJUS2022-009) and was conducted in strict accordance with the Declaration of Helsinki. The participant recruitment and screening process is illustrated in Figure 1.

**Figure 1:**
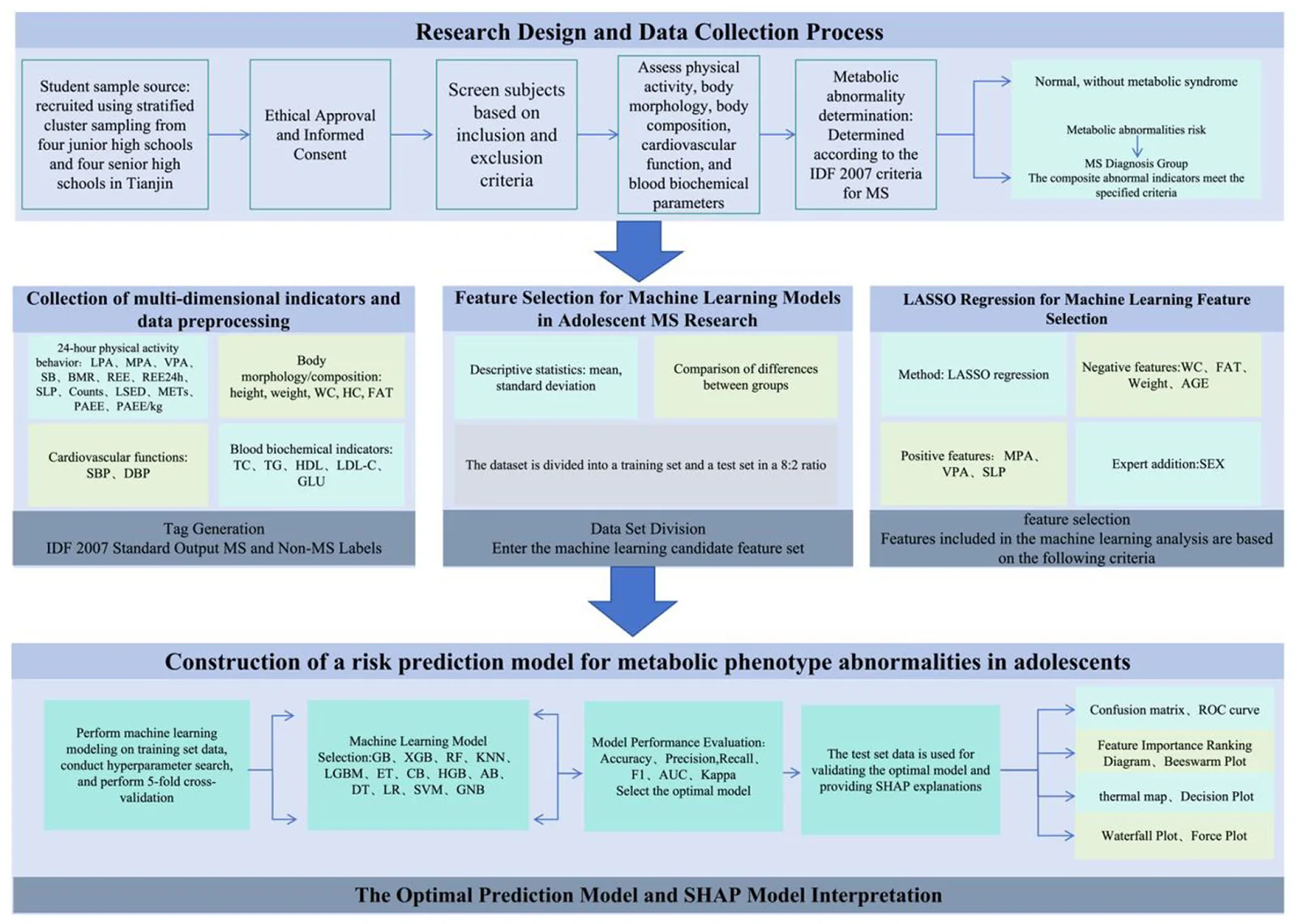
Study flowchart.

### 2.2 24-h physical activity behavior measurement

24-h activity behaviors were measured using a triaxial accelerometer (ActiGraph GT3X+, USA) (24, 25). Prior to measurement, all accelerometers were uniformly calibrated. Participants wore the accelerometer on the non-dominant wrist, positioned 1–2 cm proximal to the wrist crease, ensuring the device was firmly affixed to the skin without looseness. Participants were instructed to maintain their normal daily routines, work, and exercise patterns, avoiding deliberate increases or decreases in physical activity; they were prohibited from removing the device, submerging it in water (e.g., bathing, swimming), or exposing it to strong magnetic fields to prevent device damage or data distortion. The measurement period lasted seven consecutive days, with a minimum daily wear time of 10 h, encompassing five school days and 2 weekend days (26). Data were preprocessed using the device companion software (ActiLife 6.13.4), which removed invalid data and computed activity indices.

Extracted indicators included: light-intensity physical activity time (LPA), moderate-intensity physical activity time (MPA), vigorous-intensity physical activity time (VPA), sedentary behavior time (SB), basal metabolic rate (BMR), resting energy expenditure (REE), 24-h resting energy expenditure (REE24h), sleep duration (SLP), activity counts (Counts), leisure screen-based entertainment duration (LSED), metabolic equivalents (METs), physical activity energy expenditure (PAEE), and physical activity energy expenditure per kilogram (PAEE/kg).

### 2.3 Anthropometric measurements

#### 2.3.1 Height and weight

Height and weight were measured using a height-weight tester (Beijing Xindonghuateng Technology Development Co., Ltd., Model: GMCS-8, China). Participants stood barefoot with their back to the stadiometer column, assuming an upright standing posture on the base plate, with the trunk naturally extended and the heels, sacrum, and scapulae in contact with the column. The head was held upright, with the eyes looking straight ahead and the upper margin of the tragus and the lower margin of the orbit aligned horizontally. Height (cm) and weight (kg) were recorded, and body mass index was calculated as BMI = weight (kg)/height^2^ (m^2^).

#### 2.3.2 Waist and hip circumference

Waist circumference (WC) and hip circumference (HC) were measured using an electronic dimension meter (Beijing Xindonghuateng Technology Development Co., Ltd., Model: GMCS-8, China). Participants wore light, close-fitting clothing, stood with feet approximately 25–30 cm apart, arms hanging naturally, and maintained calm breathing in a natural standing posture. The tape measure was placed horizontally at the midpoint between the lower costal margin and the iliac crest at the mid-axillary line, with readings taken at the end of a calm expiration, accurate to 0.1 cm. Measurements were repeated twice and averaged. For hip circumference, the tape measure was placed at the widest point of the buttocks, kept horizontal to the ground, accurate to 0.1 cm, and measured twice and averaged. The waist-to-hip ratio was calculated as WHR = WC (cm)/HC (cm).

### 2.4 Body composition assessment

Body composition was assessed using dual-energy X-ray absorptiometry (DXA; Lunar iDXA, GE Healthcare, USA), the internationally recognized gold standard for body composition measurement (27, 28). Within 24 h prior to measurement, participants were required to avoid vigorous exercise and high-fat meals, and to refrain from alcohol consumption and the use of medications affecting metabolism. Prior to measurement, the instrument was fully calibrated by trained professionals. During measurement, participants removed all metallic items and wore loose, cotton clothing free of metal buttons. Participants lay supine on the scanning bed, with body posture adjusted to ensure natural extension of the head, trunk, and limbs, alignment of the body midline with the scanning bed midline, arms naturally positioned at the sides with palms facing upward, and legs together. A whole-body scan was performed, and body fat percentage (FAT) was recorded.

### 2.5 Cardiovascular function measurement

After a 15-min resting period, blood pressure was measured using an electronic sphygmomanometer (Beijing Xindonghuateng Technology Development Co., Ltd.). Participants were seated with the right upper arm naturally relaxed and abducted, with the midpoint of the upper arm at heart level and the lower edge of the cuff 2–3 cm above the antecubital fossa. Measurement was initiated once participants were emotionally stable, and measurements were taken twice consecutively with a 1-min interval, with the mean value used as the final reading, recorded to the nearest 1 mmHg. Systolic blood pressure (SBP, mmHg) and diastolic blood pressure (DBP, mmHg) were recorded.

### 2.6 Blood biochemical testing

Fasting venous blood samples (5 ml) were collected in the early morning (following 8–12 h of fasting and abstinence from sugar-sweetened beverages) into vacuum blood collection tubes containing clot activator/separation gel. After standing at room temperature for 30 min, samples were centrifuged at 3,000 rpm for 10 min (29, 30). Blood biochemical indices were determined using a fully automated biochemical analyzer (Beckman AU-2700, USA). Serum total cholesterol (TC) and triglycerides (TG) were measured using enzymatic methods; high-density lipoprotein cholesterol (HDL-C) was measured using the phosphotungstic acid-magnesium (PTA-Mg) precipitation method; low-density lipoprotein cholesterol (LDL-C) was measured using the direct method; and fasting blood glucose (GLU) was measured using the glucose oxidase method. All reagent kits were supplied by Zhongsheng Beikong Biotechnology Co., Ltd.

### 2.7 Diagnostic criteria for adolescent metabolic syndrome

MS was diagnosed according to the 2007 IDF criteria. MS was defined as the presence of elevated waist circumference (Table 1) plus any two or more of the following: elevated blood pressure, elevated fasting glucose, elevated triglycerides, or reduced HDL-C (31).

**Table 1:** Diagnostic criteria for metabolic syndrome (MS).

Age group	Waist circumference (cm)	Triglycerides (mmol/L)	HDL-C (mmol/L)	Blood pressure (mmHg)	Glucose (mmol/L)
10 ≤ 16 year	≥90th percentile	≥1.70 mmol/L	<1.03 mmol/L	SBP ≥130 or DBP ≥85 mmHg	≥5.6 mmol/L
≥16 year	Male ≥94 cm; female ≥80 cm	≥1.70 mmol/L	Male <1.03; female <1.29 mmol/L	SBP ≥130 or DBP ≥85 mmHg	≥5.6 mmol/L

### 2.8 Machine learning model construction and evaluation

Thirteen machine learning algorithms were employed to construct adolescent MS prediction models and identify the optimal model: GradientBoosting (GB), XGBoost (XGB), RandomForest (RF), K-Nearest Neighbors (KNN), LightGBM (LGBM), ExtraTrees (ET), CatBoost (CB), HistGradientBoosting (HGB), AdaBoost (AB), DecisionTree (DT), Logistic Regression (LR), Support Vector Machine (SVM), and Gaussian Naive Bayes (GaussianNB). Model hyperparameter optimization was performed using a grid search strategy, exhaustively searching for the optimal parameter combination within a predefined parameter space based on cross-validated AUC scores (32). The SMOTETomek combined sampling method was applied to resample the training set to address class imbalance (33, 34). All models were evaluated for generalization performance using five-fold stratified cross-validation (35), with stratification by the MS label to ensure the MS prevalence in each fold matched that of the overall dataset. To further assess model convergence and potential overfitting, sample-size learning curves were generated for all 13 classifiers by plotting the mean training and validation ROC-AUC across five stratified folds over increasing training sample sizes.

### 2.9 Model validation and interpretation

The best-performing model was validated on the independent test set. Comprehensive classification performance was evaluated using accuracy, precision, recall, F1 score, AUC, and the confusion matrix, with these metrics used to assess the model's predictive performance and generalizability in real-world scenarios (36, 37). SHAP values were employed to interpret the machine learning model, including feature importance ranking plots, SHAP summary (bee-swarm) plots, SHAP heatmap matrices, single-sample waterfall plots, decision path plots, and single-sample force plots.

### 2.10 Statistical analysis

Data were analyzed using SPSS 26.0 and Python 3.9 (Scikit-learn, SHAP, Matplotlib libraries). Normally distributed continuous variables are expressed as mean ± standard deviation (x̄ ± s), with between-group comparisons performed using independent samples *t*-tests or analysis of variance. Non-normally distributed variables are expressed as median, with between-group comparisons performed using non-parametric tests. Categorical variables are expressed as frequencies (percentages), with between-group comparisons performed using chi-square tests. A two-tailed *P* < 0.05 was considered statistically significant.

## 3 Results

### 3.1 Baseline characteristics of participants

A total of 822 adolescents (mean age 13 years) were enrolled, with 659 in the training set (306 males, 353 females) and 163 in the test set (79 males, 84 females). The overall MS prevalence was 8.0% (*n* = 66), with a prevalence of 8.0% (*n* = 53) in the training set and 8.0% (*n* = 13) in the test set. No statistically significant differences in MS prevalence or MS-related indicators were observed between groups (*P* > 0.05; Table 2).

**Table 2:** Baseline characteristics of participants.

Characteristic	Training set (*n* = 659)		Test set (*n* = 163)	
	Male (*n* = 306)	Female (*n* = 353)	Male (*n* = 79)	Female (*n* = 84)
Age	13.2 ± 1.0	13.1 ± 0.9	13.2 ± 1.1	13.1 ± 0.9
Height (cm)	163.5 ± 9.2	160.6 ± 8.1	165.0 ± 9.6	158.4 ± 5.9
Weight (kg)	56.5 ± 15.4	54.7 ± 14.0	59.0 ± 16.1	52.0 ± 11.2
BMI (kg/m^2^)	21.0 ± 4.6	21.2 ± 4.6	21.7 ± 5.0	20.8 ± 3.8
LPA (min/day)	237.2 ± 70.6	224.5 ± 67.1	239.7 ± 75.5	223.2 ± 61.7
MPA (min/day)	48.5 ± 22.3	44.9 ± 18.0	51.0 ± 23.4	42.4 ± 16.5
VPA (min/day)	4.1 ± 4.4	3.1 ± 3.4	4.7 ± 5.4	2.3 ± 2.1
SD (min/day)	596.6 ± 67.4	584.5 ± 71.3	597.2 ± 69.6	583.9 ± 67.0
BMR (kcal/day)	1,540.1 ± 254.3	1,456.2 ± 205.0	1,609.5 ± 259.7	1,377.3 ± 120.9
REE	1,698.4 ± 278.8	1,603.8 ± 246.7	1,786.3 ± 285.5	1,513.1 ± 145.4
REE24h	70.6 ± 11.3	66.7 ± 9.6	74.0 ± 11.5	63.2 ± 5.1
SLP (min/day)	553.6 ± 100.2	583.0 ± 99.6	547.4 ± 105.5	588.2 ± 92.6
Counts	901.4 ± 184.0	860.4 ± 156.4	936.7 ± 203.3	831.4 ± 135.7
LSED (min/day)	195.1 ± 125.6	190.7 ± 127.3	197.9 ± 124.1	179.5 ± 114.7
METs	2.5 ± 0.3	2.4 ± 0.2	2.5 ± 0.3	2.4 ± 0.2
PAEE (kcal/day)	648.6 ± 209.5	600.9 ± 193.2	678.4 ± 188.9	565.1 ± 167.9
PAEE/kg (kcal/kg/day)	11.8 ± 3.8	11.1 ± 3.2	12.3 ± 3.9	10.9 ± 2.8
HC (cm)	105.7 ± 10.0	105.7 ± 9.4	105.6 ± 10.8	105.2 ± 7.5
WHR	0.8 ± 0.1	0.8 ± 0.1	0.9 ± 0.0	0.7 ± 0.0
SBP (mmHg)	113.0 ± 13.6	111.0 ± 11.4	112.9 ± 14.6	109.4 ± 10.9
DBP (mmHg)	63.4 ± 10.6	63.1 ± 8.5	62.9 ± 11.3	62.6 ± 7.9
WC (cm)	74.0 ± 13.2	72.1 ± 12.2	75.9 ± 13.0	70.6 ± 10.2
FAT (%)	23.9 ± 10.6	27.3 ± 10.4	22.1 ± 11.1	29.3 ± 8.2
GLU (mmol/L)	4.65 ± 0.43	4.61 ± 0.45	4.60 ± 0.16	4.62 ± 0.13
TG (mmol/L)	1.00 ± 0.54	1.02 ± 0.49	1.01 ± 0.24	1.04 ± 0.25
HDL (mmol/L)	1.36 ± 0.28	1.40 ± 0.29	1.38 ± 0.11	1.38 ± 0.10
TC (mmol/L)	4.18 ± 0.68	4.31 ± 0.73	4.24 ± 0.26	4.36 ± 0.27
LDL (mmol/L)	2.36 ± 0.58	2.44 ± 0.64	2.39 ± 0.22	2.52 ± 0.21

No significant differences in baseline characteristics between training and test sets.

### 3.2 Feature selection

LASSO regression was employed for dimensionality reduction and feature selection. All candidate variables were included in the LASSO model, and the optimal regularization parameter (λ) was determined via 10-fold cross-validation, minimizing the mean squared error to select features with significant contributions to MS prediction. Eight key features were identified, among which four exhibited negative effects: WC (β = 0.21), FAT (β = 0.18), Weight (β = 0.17), and Age (β = 0.04); three features exhibited positive (protective) effects: MPA (β = −0.16), VPA (β = −0.13), and SLP (β = −0.05). Following expert consultation, sex was unanimously agreed upon as an important demographic covariate and was included in the model (Figure 2).

**Figure 2:**
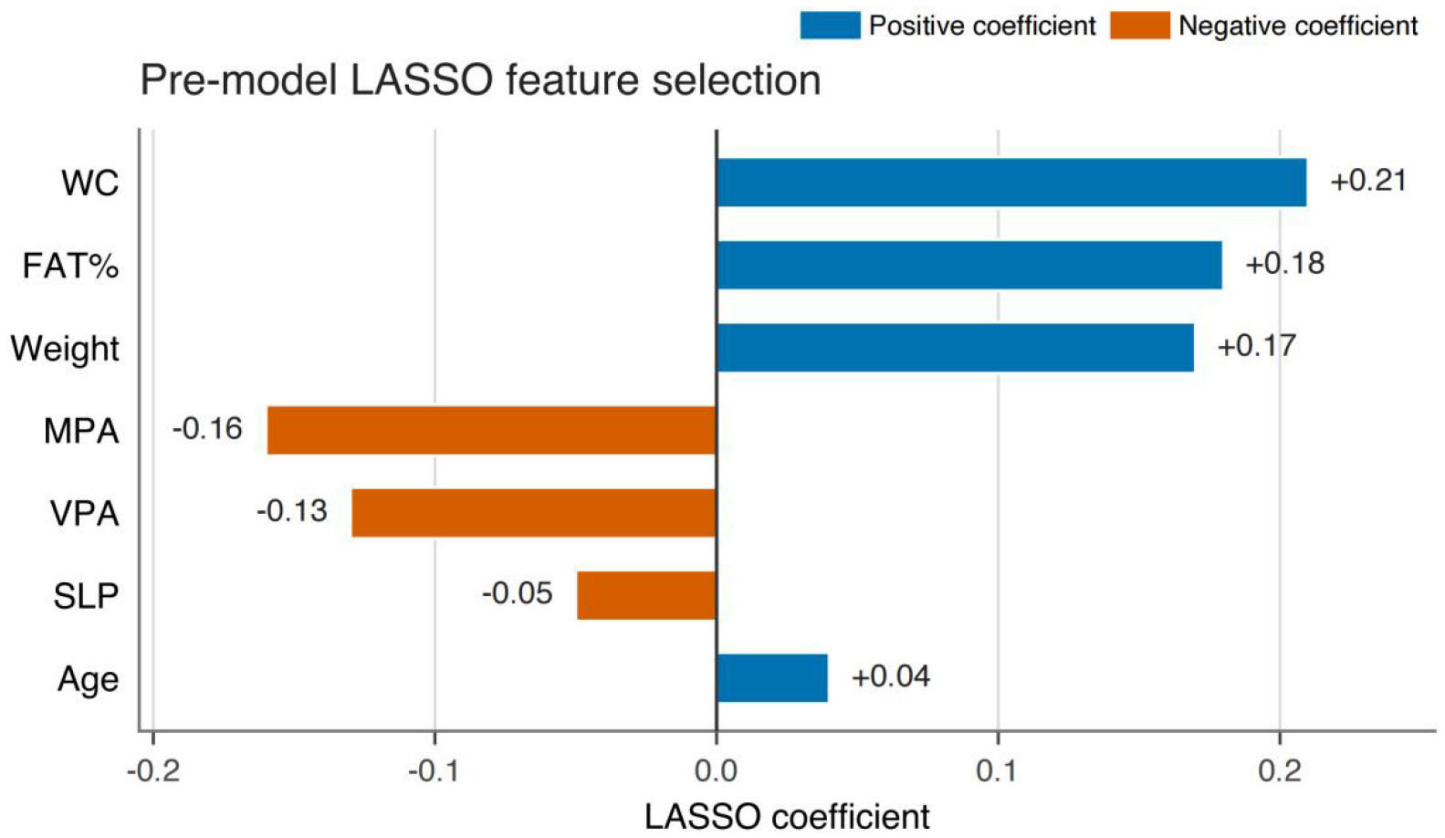
Nonzero LASSO coefficients used for pre-model feature selection. Only variables with nonzero LASSO coefficients are shown; the remaining candidate variables were regularized to zero and excluded from subsequent modeling.

### 3.3 Machine learning model construction and evaluation

Thirteen machine learning classification models were constructed, and model performance was systematically evaluated using five-fold cross-validation. The results showed that the Extra Trees model achieved the highest AUC (0.749 ± 0.043) and an F1 score of 0.798 ± 0.028. Integrating multiple evaluation metrics including accuracy, AUC, and F1 score, the Extra Trees model demonstrated the most balanced performance and was selected as the optimal model for adolescent MS risk prediction (Table 3, Figure 3). The learning-curve analysis revealed heterogeneous training behavior across the 13 classifiers. Extra Trees showed the most pronounced and consistent improvement in both training and cross-validation ROC-AUC as the training sample size increased. AdaBoost exhibited a weaker upward trend, whereas DecisionTree and LogisticReg showed non-monotonic or declining training trajectories with only modest improvement in cross-validation performance. The remaining classifiers, including XGBoost, RandomForest, KNN, LightGBM, GradientBoosting, CatBoost, HistGradientBoosting, SVM, and GaussianNB, showed relatively flat or only modestly changing training trajectories, indicating limited incremental learning under the present sample-size and feature configuration. Sample-size learning curves for all 13 classifiers are presented in Supplementary Figure S2.

**Table 3:** Model evaluation results of 5-fold cross-validation.

No.	Model	Accuracy	Precision	Recall	F1	Kappa	AUC
1	ExtraTrees	0.838 ± 0.022	0.836 ± 0.017	0.837 ± 0.022	0.798 ± 0.028	0.259 ± 0.082	0.749 ± 0.043
2	XGBoost	0.838 ± 0.013	0.816 ± 0.015	0.831 ± 0.013	0.791 ± 0.020	0.253 ± 0.109	0.695 ± 0.049
3	RandomForest	0.831 ± 0.023	0.836 ± 0.022	0.835 ± 0.021	0.796 ± 0.029	0.254 ± 0.105	0.735 ± 0.049
4	KNN	0.822 ± 0.027	0.804 ± 0.034	0.822 ± 0.027	0.796 ± 0.038	0.284 ± 0.057	0.709 ± 0.041
5	LightGBM	0.830 ± 0.024	0.792 ± 0.066	0.830 ± 0.024	0.791 ± 0.024	0.247 ± 0.141	0.691 ± 0.057
6	GradientBoosting	0.836 ± 0.030	0.816 ± 0.033	0.836 ± 0.030	0.797 ± 0.034	0.310 ± 0.075	0.706 ± 0.054
7	CatBoost	0.830 ± 0.025	0.826 ± 0.010	0.830 ± 0.025	0.784 ± 0.034	0.217 ± 0.051	0.711 ± 0.062
8	HistGradientBoosting	0.822 ± 0.031	0.809 ± 0.037	0.822 ± 0.031	0.783 ± 0.062	0.367 ± 0.053	0.670 ± 0.044
9	AdaBoost	0.826 ± 0.004	0.837 ± 0.048	0.826 ± 0.004	0.769 ± 0.059	0.259 ± 0.174	0.709 ± 0.054
10	DecisionTree	0.796 ± 0.049	0.757 ± 0.057	0.796 ± 0.049	0.765 ± 0.052	0.111 ± 0.167	0.598 ± 0.067
11	LogisticReg	0.816 ± 0.042	0.781 ± 0.054	0.816 ± 0.042	0.765 ± 0.059	0.165 ± 0.060	0.638 ± 0.101
12	SVM	0.824 ± 0.041	0.846 ± 0.035	0.824 ± 0.041	0.761 ± 0.055	0.139 ± 0.124	0.727 ± 0.064
13	GaussianNB	0.754 ± 0.023	0.736 ± 0.029	0.754 ± 0.023	0.758 ± 0.048	0.274 ± 0.213	0.651 ± 0.076

**Figure 3:**
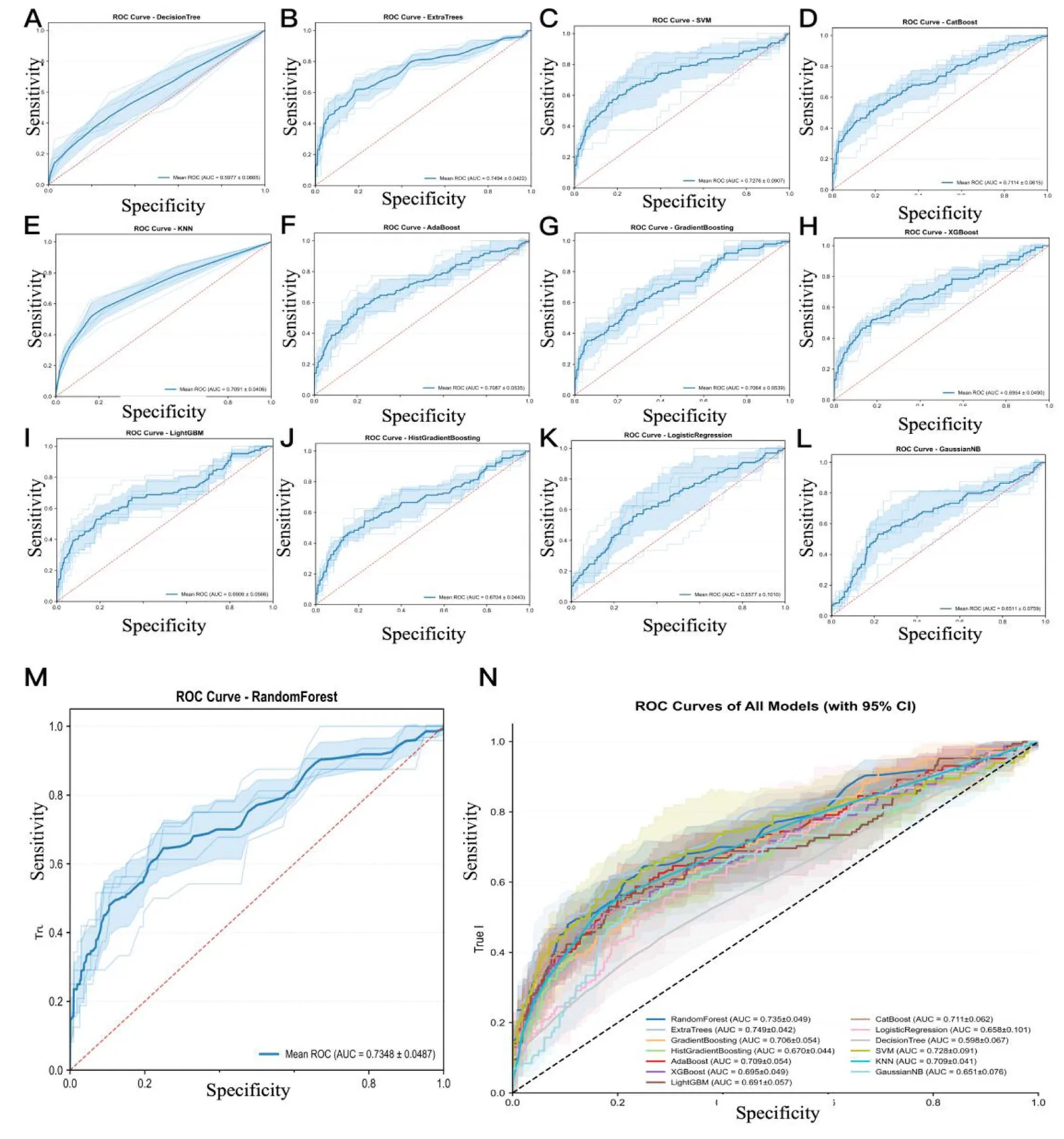
ROC curves of the 13 machine-learning classifiers. **(A)** Decision Tree; **(B)** Extra Trees; **(C)** support vector machine (SVM); **(D)** CatBoost; **(E)** k-nearest neighbors (KNN); **(F)** AdaBoost; **(G)** Gradient Boosting; **(H)** XGBoost; **(I)** LightGBM; **(J)** Histogram-based Gradient Boosting; **(K)** Logistic Regression; **(L)** Gaussian Naive Bayes; **(M)** Random Forest; and **(N)** comparison of the ROC curves of all 13 classifiers with 95% confidence intervals.

### 3.4 Validation of the optimal model

The hyperparameter-optimized Extra Trees model correctly identified 140 MS-negative individuals and 10 MS-positive individuals in the test set, with 10 false positives and three false negatives. Model performance evaluation revealed an accuracy of 92.02%, precision of 50.00%, sensitivity (recall) of 76.92%, F1 score of 60.61%, and Cohen's Kappa coefficient of 0.5639, indicating moderate concordance between predicted outcomes and actual diagnoses. The area under the ROC curve (AUC) reached 0.8638, demonstrating good comprehensive discriminative capability (Figures 4a, b).

**Figure 4:**
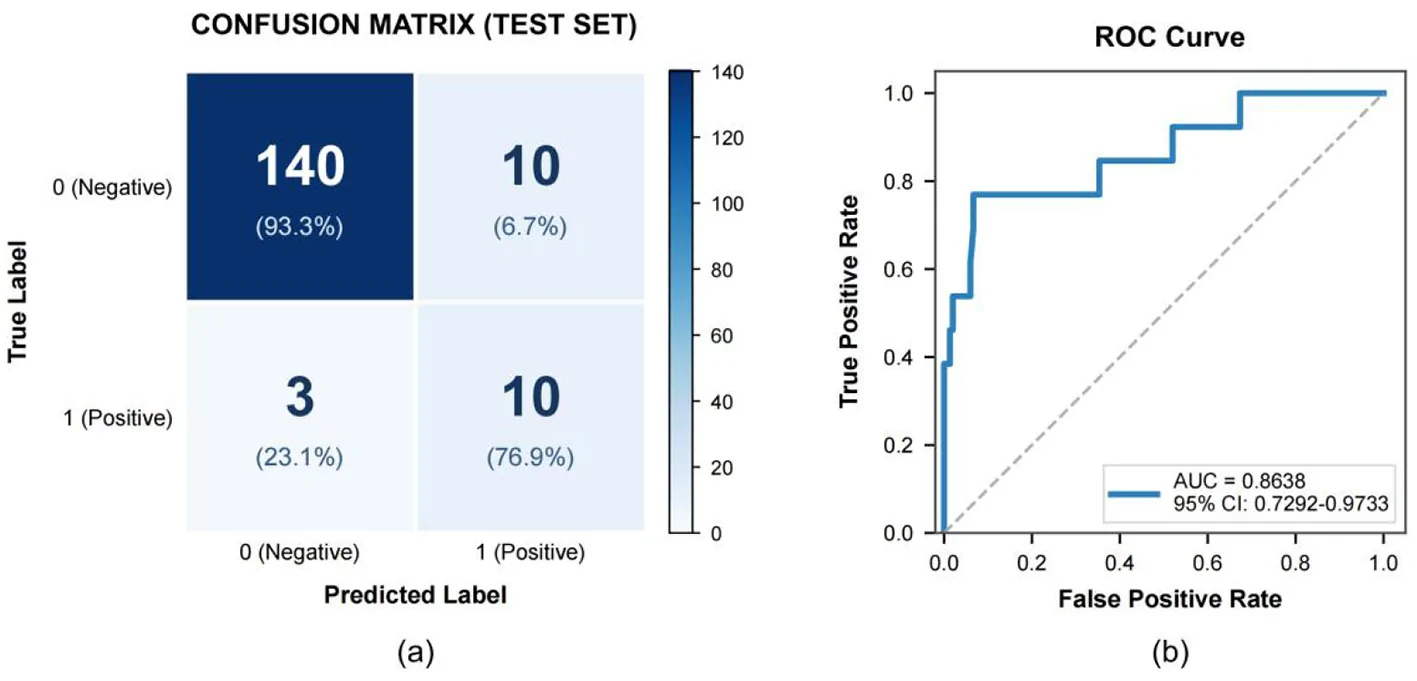
Performance evaluation of the hyperparameter-optimized Extra Trees model in the held-out internal test set: **(a)** confusion matrix and **(b)** receiver operating characteristic (ROC) curve.

### 3.5 Model interpretation

SHAP values were employed to interpret the decision-making mechanisms of the Extra Trees model. Feature importance ranking revealed the following order (Figure 5a): FAT (SHAP = 0.08), Weight (SHAP = 0.04), MPA (SHAP = 0.04), WC (SHAP = 0.03), VPA (SHAP = 0.03), SLP (SHAP = 0.02), and Age (SHAP = 0.02).The SHAP summary (bee-swarm) plot (Figure 5b) showed that FAT was the feature with the greatest contribution to MS prediction, with SHAP values distributed across −0.05 to 0.28; Weight (−0.08 to 0.20), WC (−0.06 to 0.18), and MPA (−0.06 to 0.15) exhibited similar positive trends. The SHAP heatmap (Figure 5c) revealed that FAT was predominantly red across most samples (SHAP range: +0.05 to 0.20), with decreasing intensity for Weight, WC, and MPA, while SLP, Age, and Sex contributed less.The waterfall plot (Figure 5d) for the first sample showed that Age (SHAP = −0.09) and WC (SHAP = −0.05) provided the largest negative contributions, followed by MPA (+0.03), VPA (+0.03), and Weight (+0.03) providing positive predictive contributions, indicating that protective features can dominate the final prediction direction. The decision plot (Figure 5e) demonstrated that features were arranged top-down by importance, with sample prediction probabilities diverging from a baseline of 0.20; high-risk samples (red) reached final probabilities exceeding 0.40, whereas low-risk samples (blue) fell below 0.10. The force plot (Figure 5f) indicated that the positive driving factors included FAT = 0.464, Weight = 0.041, MPA = −0.440, and VPA = 0.850, with FAT exerting the largest positive force; the negative driving factors were Age = 1.843 and WC = −0.169. To further enhance the clinical interpretability of the model predictions, partial dependence analysis was performed for the leading predictors; the two-way partial dependence response surface for WC and FAT% is provided in Supplementary Figure S1. Additionally, native feature importance of the final Extra Trees classifier was quantified using normalized mean decrease in impurity (Gini importance), and the resulting feature-importance ranking is presented in Supplementary Figure S3.

**Figure 5:**
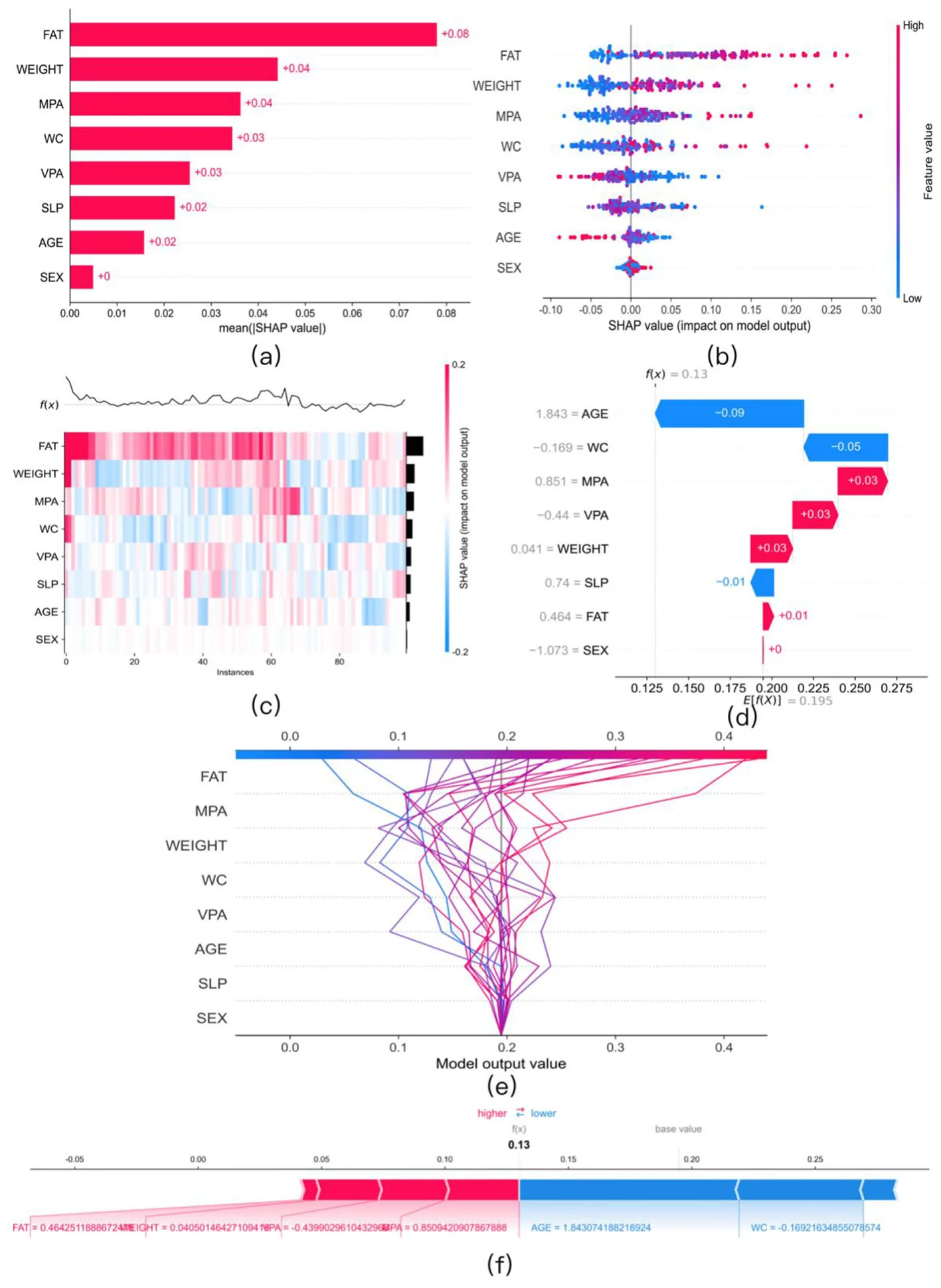
SHAP-based interpretation of the Extra Trees model for predicting metabolic syndrome in adolescents: **(a)** global feature-importance plot based on mean absolute SHAP values; **(b)** SHAP summary (beeswarm) plot; **(c)** SHAP heatmap across participants; **(d)** waterfall plot for an individual prediction; **(e)** decision plot across individual predictions; and **(f)** force plot for an individual prediction.

## 4 Discussion

The prevalence of MS among adolescents continues to rise globally and has become a critical public health concern threatening long-term cardiovascular health in youth. 24-h activity behaviors—including sleep, sedentary behavior, and physical activity of varying intensities—were examined as compositional predictors of adolescents' metabolic health. This study established a machine learning-based adolescent MS prediction model and identified, via LASSO feature selection, WC (β = 0.21), FAT (β = 0.18), Weight (β = 0.17), and Age (β = 0.04) as predictors associated with MS classification. The IDF 2007 criteria designate central obesity as a mandatory component for adolescent MS diagnosis (31). WC serves as a direct surface-level proxy for abdominal fat accumulation, particularly visceral adipose tissue, and is anatomically proximate to the visceral fat depot (38), suggesting that WC can distinguish between metabolic phenotypes with identical BMI but markedly different fat distributions; for instance, two adolescents with the same BMI may exhibit substantially different visceral fat burdens and cardiometabolic risk profiles (39). A systematic review further confirmed that WC is more strongly associated with atherogenic dyslipidemia, insulin resistance, alanine aminotransferase levels, and serum uric acid than BMI (40). Visceral adipocytes exhibit high lipolytic rates, releasing large quantities of free fatty acids into the portal circulation, which directly impairs hepatic insulin signaling, enhances gluconeogenesis, and promotes excessive very-low-density lipoprotein synthesis, which have been proposed as biological pathways linking visceral adiposity with dyslipidemia and insulin resistance (41). FAT is a core body composition indicator for assessing individual fat accumulation. Olafsdottir et al. (42) measured body fat percentage in 182 18-year-old adolescents and found that 42% of those with normal BMI had elevated body fat, indicating the presence of normal weight obesity (NWO). These adolescents had an odds ratio of 2.2 (95% CI: 1.2–3.9) for having one or more MS risk factors, and four of the five adolescents diagnosed with MetS were classified as NWO. Although Zhang et al. (20) achieved high predictive performance (AUC = 0.969) for adolescent MS using NHANES data, their model did not include body fat percentage, which may systematically underestimate metabolic risk in NWO-phenotype adolescents with distinct body fat compositions at equivalent BMI levels. This study further identified MPA (β = −0.16), VPA (β = −0.13), and SLP (β = −0.05) as protective factors against adolescent MTS. The WHO 2020 guidelines, for the first time, integrated moderate-to-vigorous physical activity and adequate sleep into a unified 24-h activity behavior recommendation framework, explicitly stating that ≥60 min of daily MVPA and 9–11 h of adequate sleep confer independent protective effects on cardiometabolic health in children and adolescents (12). Spaulding and Yan (43) demonstrated that MPA and VPA improve peripheral tissue glucose uptake and utilization through the AMPK-PGC-1α-centered metabolic regulatory cascade. A systematic review by Poitras et al. (44) confirmed that insufficient MVPA is significantly and independently associated with individual MS components in adolescents, including elevated waist circumference, elevated blood pressure, reduced HDL-C, and impaired glycemic regulation. In the present study, the effect size of MPA (β = −0.16) exceeded that of VPA (β = −0.13), suggesting that the marginal protective effect of MPA may be greater than that of VPA for adolescents. Karchynskaya et al. (45) noted that, given the developmental characteristics of adolescents, VPA has higher participation thresholds and stronger motor skill demands, whereas cardiorespiratory fitness in adolescents is not yet fully mature; in contrast, MPA activities such as brisk walking, jogging, and cycling are more accessible and amenable to sustained engagement. Che et al. (46) found that insufficient SLP promotes MS component development through multiple pathways, including sympathetic overactivation, disrupted circadian cortisol rhythms, dysregulated appetite hormones, and systemic chronic inflammation. Furthermore, LPA and SB were not retained, which may be attributable to the fact that, within a compositional data framework, MPA and VPA already possess stronger independent discriminative power, and the marginal contributions of LPA and SB are subsumed. The compositional isotemporal substitution model proposed by Dumuid et al. (14) posits that, under the fixed 24-h time constraint, competitive substitution relationships exist among components, with LPA and SB influencing health primarily through indirect pathways involving the displacement of MPA and VPA.

The systematic comparison of 13 machine learning algorithms revealed that Extra Trees demonstrated the most favorable comprehensive discriminative performance in five-fold cross-validation (Table 3, Figure 3) and exhibited good accuracy, specificity, and AUC, with moderate agreement according to Cohen's Kappa, in the internal test set (Figure 4). Although the model achieved an overall accuracy of 92.02%, its sensitivity was lower at 76.92%, indicating that three of the 13 adolescents with MS were not identified. Given the low MS prevalence (8.0%), overall accuracy was influenced by the predominance of MS-negative participants and should not be interpreted in isolation. In a screening context, prioritizing sensitivity may be appropriate even at the cost of more false-positive referrals; however, the precision of 50.00% indicates that screen-positive adolescents would require confirmatory clinical assessment. The comparatively favorable learning pattern and predictive performance of Extra Trees may be attributable to its ability to capture nonlinear threshold effects and interactions among adiposity and 24-h movement variables without requiring prespecified functional forms. These properties may explain why Extra Trees showed a more pronounced response to increasing training sample size than the other classifiers (47). The advantage of Extra Trees lies in its algorithm-level dual randomization splitting strategy: the split threshold for each candidate feature is selected entirely at random. This “feature randomization + threshold randomization” dual mechanism slightly increases the bias of individual decision trees but substantially reduces variance (48, 49). Chen et al. (50) demonstrated that Bayesian hyperparameter-optimized Extra Trees achieved high predictive performance (AUC = 96.61%, accuracy = 91.80%) in ICU mortality risk prediction, effectively suppressing overfitting under conditions of high-dimensional small samples and severe class imbalance (ICU mortality rate approximately 10%−15%). Arslan and Yapici (51) similarly reported that Extra Trees combined with SMOTE achieved globally optimal performance (AUC = 97.99%, accuracy = 91.93%) in an obesity classification task involving 1,610 adults, exhibiting stable decision boundaries when handling imbalanced data. Nevertheless, comparative evidence suggests that the relative performance of machine-learning algorithms is context dependent. Shin et al. (52) compared logistic regression, decision tree, random forest, XGBoost, and TabNet for non-invasive MS prediction; although XGBoost achieved the highest optimized AUC (0.893), a simpler decision-tree model was selected for the final application because it used fewer readily obtainable predictors and achieved a comparable AUC (0.889). In a pediatric cohort, Choi et al. (53) reported that XGBoost performance varied according to predictor composition, with AUCs of 0.56, 0.61, and 0.74 for anthropometric, bioelectrical impedance, and combined models, respectively, in a real-world validation dataset. These findings indicate that no single algorithm can be assumed to be uniformly optimal, and that the favorable performance of Extra Trees in the present study should be interpreted in the context of the study population, predictor set, and validation design. This may be because SMOTETomek, through SMOTE-based synthesis of minority class samples to enhance positive class representativeness and Tomek Links-based removal of boundary noise to optimize decision boundaries, mitigates class bias while avoiding the information loss inherent in pure oversampling or undersampling approaches (54). The MS prevalence of 8.0% in this study is comparable to the 4.2%−9.2% prevalence reported by Ferranti et al. (7) for American adolescents. SHAP value analysis revealed the following ranking of predictive features by contribution to MS risk: FAT, Weight, MPA, WC, VPA, SLP, and Age (Figures 5a, b). FAT exerted the greatest influence, with high-FAT samples concentrated in the positive SHAP region, exhibiting a monotonic positive correlation with MS risk. This suggests that excessive adipose tissue during adolescence continuously secretes pro-inflammatory cytokines and reduces adiponectin levels, inducing insulin resistance, which in turn leads to compensatory hyperinsulinemia, impaired glucose regulation, dyslipidemia, and elevated blood pressure. Beyene et al. (55) proposed adjusting BMI using biochemical markers to differentiate metabolic health status under identical BMI values, validating the limitations of BMI in reflecting metabolic heterogeneity; the present SHAP results, from a machine learning interpretability perspective, similarly confirm that FAT is a critical predictive feature. The waterfall plot (Figure 5d) showed that Age (SHAP = −0.09) and WC (SHAP = −0.05) provided the largest negative contributions. Marques et al. (56), in a network analysis of obese adolescents, demonstrated that from early to late puberty, visceral fat, fat mass, and waist circumference increased significantly (P <0.001), and the association structure among cardiometabolic risk factors underwent qualitative changes with sexual maturation stage. This is consistent with the puberty-specific insulin resistance mechanisms reviewed by Luo et al. (57), wherein activation of the GH/IGF-1 axis and reorganization of sex hormone levels jointly induce a transient decline in insulin sensitivity, providing the endocrinological basis for the progressive accumulation of metabolic abnormalities. Importantly, the negative SHAP values for Age and WC in the selected sample represent local contributions relative to the model baseline rather than protective population-level effects (36). The negative contribution of WC may reflect the participant's value relative to the model reference, whereas the negative contribution of Age may result from the restricted age range, nonlinear interactions with adiposity-related variables, and heterogeneity in pubertal maturation (56, 57). Therefore, these findings do not contradict the established epidemiological roles of age and WC as risk factors for MS (31, 58). The force plot (Figure 5f) further illustrated the sample-specific nature of the prediction, with Age and WC producing the largest downward shifts relative to the baseline. This suggests that, for adolescents with elevated FAT and low MPA, interventions should simultaneously target “increasing activity” and “reducing body fat,” whereas for those with elevated WC but some level of MPA engagement, interventions should focus on reducing visceral adiposity, thereby enabling precision prevention and control of adolescent MS. To complement the SHAP-based interpretation, native Gini importance ranked WC first. The relatively higher ranking of WC in the native importance measure may be attributable to two factors. First, WC is closely related to the central-obesity component required by the 2007 IDF definition of adolescent metabolic syndrome; consequently, threshold-based splits on WC may produce a relatively large reduction in node impurity. Second, WC, FAT%, and Weight are correlated indicators of adiposity. Native Gini importance may accumulate the gains from tree splits attributed to WC, whereas SHAP may distribute the predictive information shared by these correlated predictors at the level of individual predictions. Thus, the native importance ranking provides complementary support for the importance of adiposity-related predictors; however, the higher native importance of WC should not be interpreted as evidence of a stronger independent predictive contribution of WC (59).

### 4.1 Limitations

Several limitations should be acknowledged. First, the cross-sectional design precludes determination of temporal ordering and causal relationships. Second, all participants were recruited in Tianjin, China; the recruitment and participation processes may have introduced selection bias, thereby limiting the generalizability of the findings. Third, model validation was limited to stratified five-fold cross-validation and a held-out internal test set, and no independent cohort from another region was used for external validation. Therefore, the external validity and transportability of the model remain to be established. Moreover, because model selection was based on the same cross-validation framework, the two-way split may have introduced some optimism into the internal performance estimates; a three-way training–validation–test split would be preferable when the sample size permits. Fourth, the predictors included in this study were predominantly non-invasive indicators and did not include serum biochemical markers, which may limit the model's sensitivity for detecting some early metabolic abnormalities. Finally, because the model is intended for large-scale screening, the cost and accessibility of DXA, as well as the availability and use of accelerometers, may limit its practical implementation. Bioelectrical impedance analysis and questionnaires may serve as lower-cost alternatives for body-composition and physical-activity assessment, respectively, but their reliability and validity should be verified against DXA and accelerometer measurements before adoption.

### 4.2 Future research

Future studies should prioritize multicenter external validation across different regions and ethnic populations, prospective cohort studies to assess the temporal performance and generalizability of the model, and direct comparisons with deep-learning approaches. Further research should also evaluate real-world implementation, including feasibility, cost-effectiveness, workflow integration, acceptability, and screening performance in clinical, school, and community settings.

## 5 Conclusions

This study developed a machine-learning model for adolescent metabolic syndrome prediction that integrates 24-h movement behaviors with anthropometric indicators. Among the evaluated algorithms, Extra Trees showed the most favorable performance in the internal validation analyses. SHAP analysis identified FAT% as the most influential predictor and MPA as the leading movement-behavior predictor, improving model transparency and potentially enhancing clinician confidence in interpreting individual predictions. The model may provide a convenient tool for preliminary MS risk stratification in school and community settings; however, its suitability for large-scale health screening should be confirmed through multicenter external validation. These predictive associations may inform prevention strategies emphasizing physical activity and body-fat management but should not be interpreted as causal effects.

## Data Availability

The raw data supporting the conclusions of this article will be made available by the authors, without undue reservation.
