# Supplementary material for: Predicting metabolic syndrome in adolescents from device-measured 24-h movement behaviors: a machine learning approach
Source: Front Public Health. 2026 Sep 17;14:1928562. doi: 10.3389/fpubh.2026.1928562 (PMC13627327; doi:10.3389/fpubh.2026.1928562)
Supplement: Supplementary file 1 [file Table_1.docx]

**Supplementary Table S1. Class Distribution Before and After SMOTETomek Resampling**

| Dataset | Condition | MS+ | MS− | Total | MS+ proportion |
| --- | --- | --- | --- | --- | --- |
| Training | Before resampling | 53 | 606 | 659 | 8.0% |
| Training | After SMOTE | 606 | 606 | 1212 | 50.0% |
| Training | After SMOTETomek | 588 | 588 | 1176 | 50.0% |
| Test | Unchanged | 13 | 150 | 163 | 8.0% |

Note: Resampling was applied only to the training set; the test set remained unchanged.
